# The Cyclopædia of Practical Surgery, Comprising a Series of Original Dissertations on Operative Medicine, by an Association of Physicians and Surgeons

**Published:** 1837-07

**Authors:** 


Art. IV.—*The Cyclopædia of Practical Surgery, comprising a Series of Original Dissertations on Operative Medicine, by an Association of Physicians and Surgeons.* Edited by W. B. Costello, m.d., Member of several learned Societies, both National and Foreign. Published in Parts every alternate month. Part I.—*London,* 1837. Royal 8vo. pp. 112.

The press of other matter has not left us sufficient space, in the present Number, to notice the work before us at any length; but we cannot let a whole quarter pass without calling the particular attention of our readers to it. It is another of those splendid literary undertakings for which the profession is indebted to the spirit and liberality of our own worthy publishers, and is got up on precisely the same plan and in the same form as the two admirable works which have preceded it, the Cyclopædia of Practical Medicine and the Cyclopædia of Anatomy and Physiology. The array of talent combined in its execution is, if possible, still greater than on the former occasions; at least, the contributors are much more numerous, (nearly ninety,) and the list includes the name of almost every distinguished surgeon in this country, as well as a few of the most eminent in France and Germany. Judging from these premises, rather than from the contents of the First Part, which contains only the articles from *Abdomen* to *Ambulance*, we have no doubt that the work will equal its predecessors in scientific and practical value, and do honour to the surgeons and surgery of this country.

As the work proceeds, we shall feel it to be our duty to notice, from time to time, its contents, and probably to compare these with similar articles in works of the same class now publishing in Germany and France. We trust we shall have no reason to fear the comparison; but we must confess that some of the articles in the present Part are not exactly such as we could wish to enter the field with, in a contest with our scientific brethren on the continent. We must also take this opportunity of protesting, in the strongest manner, against the fashion which the editor seems disposed to adopt, of making use of a *French* or *Frenchified* nomenclature and style, altogether uncalled for, and therefore unjustifiable. If we are rich enough in surgical science to produce an English Cyclopædia of Surgery, surely our language is rich enough to dispense with foreign words, more especially words borrowed from a tongue so confessedly poor as the French. We quarrel especially with Dr. Costello's title, "*Operative Medicine,*" which we maintain to be not at all English, and not very good French, although a literal translation of the "Médecine Operatoire" of our neighbours. According to our reading of the phrase, we would say that it applied much better to a dose of jalap than to a system of Surgery. To say nothing of the table of contents of the work prefixed to this Part, in which we find a vast number of new and unnecessary terms from the French school, which look doubly barbarous in their English garb, we would ask Dr. Costello what such terms as *Ablation*,* *Accident* (symptom), *Adustion*, *Aide* (assistant surgeon), *Alese* (a spare sheet folded under a bed-ridden patient,) *Alveoles* (sockets of the teeth, *alveoli*), &c. have to do in an *English* Dictionary of Surgery?

* In this short article, which the editor has translated almost verbatim from the Dictionnaire de Médecine, the author informs us that Hippocrates applied the term to any sort of evacuation. This, we find, is taken from the original statement in Castelli's Lexicon, (art. *Ablatio*,) and is so far true as applied to the Greek synonym ἀφαίρεσις; but it surely sounds somewhat oddly, in defining the meaning of a Latin word, to refer to a Greek author.

